# Use of simulation models when developing and testing hospital evacuation plans: a tool for improving emergency preparedness

**DOI:** 10.1186/s13049-023-01105-w

**Published:** 2023-08-29

**Authors:** Monica Rådestad, Cecilia Holmgren, Ellinor Linde Blidegård, Kristina Lennquist Montán

**Affiliations:** 1 Department of Clinical Science and Education, Södersjukhuset, Karolinska Institutet, Sjukhusbacken 10, Stockholm, SE-118 83 Sweden; 2 Capio S:t Görans sjukhus, Sankt, Göransplan 1, Stockholm, SE-112 81 Sweden; 3 Södersjukhuset, Sjukhusbacken 10, Stockholm, SE-118 83 Sweden; 4 Department of Global Public Health, Karolinska Institutet, Stockholm, SE-171 77 Sweden

**Keywords:** Decision making, Emergency planning, Emergency preparedness, Hospital evacuation, Simulation exercise

## Abstract

**Background:**

In recent decades, analyses of hospitals evacuations have generated valuable knowledge. Unfortunately, these evacuation case studies often lack crucial details and policies that would be helpful in evacuation preparedness. The aim of this study was to use a simulation model to illustrate how it can aid emergency planners in the development, testing, and revising of hospitals evacuation plans. This study includes evacuation exercises at two emergency hospitals in Region Stockholm, Sweden.

**Methods:**

A scientifically validated simulation system for “table top” exercises was used for interactive training of hospital medical staff, prehospital staff and collaborating agencies. All participants acted in their usual professionals’ roles. The exercises were run in real-time and mirrored actual hospital resources with the aid of moveable magnetic symbols illustrating patients, staff and transport, presented on whiteboards. During the exercises, observers and independent instructors documented actions taken and post-exercise surveys were conducted to obtain reactions and compare results.

**Results:**

The simulation system allowed the emergency planner to test the whole evacuation process, making it possible to train and evaluate the important functions of management, coordination, and communication. Post-exercise surveys explored participants perception of the exercises. Analysis of open-ended questions included areas for improvement and resulted in five main categories: (1) management and liaison; (2) communication; (3) logistics; (4) medical care and patient prioritisation; and (5) resource utilisation.

**Conclusions:**

This study has shown that “table top” exercises using a validated simulation system can serve to guide emergency planners when developing evacuation plans, procedures, and protocols as well in training of all medical staff. The system also served to train adaptive thinking, leadership, communication, and clarification of critical functions.

## Background

Until recently, disaster preparedness, and hospital-based exercise training have focused on the medical response to high influx of patients as a result of major incidents e.g., in connection to terrorist attacks, major trauma, or patients exposed to hazardous materials [1, 2]. Several events have emphasised the need for hospitals to have an “all-hazards” approach when planning for response capacities [3, 4]. Review of the literature shows that fires and sudden-onset incidents (e.g., earthquakes, floods, and hurricanes) appear to be the usual causes for hospital evacuation [2, 5–11] but an “all hazards” approach also includes crises such as pandemics, the threat of cyberterrorism, actions of war, and failure of hospital infrastructure (e.g., electric power, water supply, and technology systems) and should be considered a threat equal to that of high patient surge situations [11, 12]. It is crucial that healthcare authorities, Emergency Medical Services (EMS) administrators, medical directors and emergency planners consider this approach in preparedness and in planning for evacuation of hospitals [5, 9, 13, 14].

In recent decades, analyses of hospitals evacuations have generated valuable knowledge, most importantly the need to test local evacuation plans before a disaster occurs [2, 5, 7, 8, 15, 16]. Unfortunately, these evacuation case studies often lack crucial details and policies that would be helpful for emergency planners when developing feasible procedures, checklists, and planning for evacuation drills [2, 5, 7, 15]. In Sweden, two models for interactive training (learning by doing) in emergency preparedness is commonly used, practical field exercises or “table top” exercises. Both models have certain advantages or disadvantages. A challenge for emergency planners is to choose the most suitable model to achieve the best learning outcomes for participants. Drills must be conducted to ensure that the evacuation plan is workable. This study suggests that simulation exercises creating an authentic environment for active learning through practice and reflection could facilitate this process [17, 18]. As the use of simulation exercises is commonly used in emergency preparedness, the need for validated simulation models has become more important.

In order to reduce the vulnerability of hospitals to major incidents and to maintain healthcare capacity regardless the type of hazard that occurs, Swedish regional authorities have the overall responsibility for crisis and disaster preparedness in their geographical area, including evacuation [19]. Until now, Sweden has experienced few hospital evacuations in comparison to other countries, and evacuation planning at most Swedish hospitals has been either inadequate or infrequently tested [1]. The exercises in this study took place at two emergency hospitals in Region Stockholm, Sweden. Region Stockholm is the largest healthcare provider in Sweden with six emergency hospitals, three pediatric emergency wards, ten local emergency clinics, and nine minor hospitals. Its main responsibility is for the society’s publicly funded healthcare. Region Stockholm includes Stockholm County, with 26 primary municipalities, and a population of 2.4 million inhabitants which is over 20% of the total Swedish population. In terms of area, Stockholm County belongs to one of the smaller counties in Sweden (approximately 6.500 square kilometers). All hospitals in Region Stockholm have a plan for surge capacity, but until recently lacked evacuation plans. The lack of standard guidelines for evacuation is recently highlighted in a literature review concerning evacuation preparedness in hospitals within the European Union (EU) and non-EU countries [11].

The aim of this study was to use a simulation system to illustrate how it can aid emergency planners in the development, testing, and revising of hospitals evacuation plans.

## Materials and methods

### Study design and setting

In this study the MAss Casualty SIMulation system, MACSIM®, a standardized model for “table top” exercises, was used to train hospital staff in medical response and decision making in an evacuation scenario. The system is a scientifically validated system developed for education and training, methodological research and development, and quality assurance in disaster medicine. An international group of experts in Disaster Medicine developed the system in 2009 and it has been used to train more than 5000 health care professionals, but also rescue service, police, and military personnel [20–25].

The MACSIM system is a didactical tool as well as a method for testing capacity and preparedness of hospitals in reponse to a simulated event. The advantage of simulation exercises is to train the whole chain of management simultaneously to illustrate real conditions during an evacuation process. The exercises described in this study are based on the simulation system and performed in a controlled environment with a counterplay. These kind of exercises are run in real-time and mirrors actual hospital resources with the aid of moveable magnetic symbols to illustrate patients, staff, and transport. The symbols are presented on magnetic whiteboards. The participants were selected based on their profession, availability, and willingness to take part. They were expected to work actively in their own roles, making it possible to train and evaluate decisions made.

The regional authorities’ regulations and guidelines require hospitals to perform regular exercises, including specific types of hazards that require evacuation. This was the first time the simulation system was used to analyse and identify issues in the evacuation process. Prior to the exercise, participants had the opportunity to participate in several workshops where the system and its components were explained. Exercise 1 was organised to provide a basis for the development and establishment of hospital evacuation plans in Region Stockholm. In 2019 evacuation plans were implemented at all hospitals in the region. Exercise 2 was conducted in order to test application of the evacuation plan, using the same simulation system and scenario as in Exercise 1. The scenario involved an infrastructure failure that resulted in prolonged loss of municipal water supply necessitating total hospital evacuation. In the scenario the term “evacuation” means the need to transfer patients to other medical facilities or to be discharged. The hospital in Exercise 1 had 620 beds, around 4100 staffs and served 600 000 inhabitants, and the hospital in Exercise 2 had 302 beds, over 1800 staffs and served 420 000 inhabitants.

### Participating functions

The following functions participated in the exercise: hospital property management, hospital security, and hospital incident command group (HICG), medical staff from affected units participated including ambulatory care, emergency department (ED), intensive care unit (ICU), maternity-, medical-, neonatal-, neurology-, obstetrics/gynecology-, oncology-, orthopedic-, and pediatric departments, post-critical care, and surgical units. Also collaborating agencies and staff working within the evacuation chain outside the hospital: EMS, police, rescue service, and military participated. During the exercises, observers and independent instructors documented actions taken (e.g., decision making, the use of protocols, response times, and observations of transport logistics). Specific data regarding participants is presented in Table 1.

**Table 1:** Different categories of participating staff

	Exercise 1	Exercise 2
Profession	n (%)	n (%)
Hospital incident command group	24 (16.6)	20 (21)
Physicians	13 (8.9)	9 (9)
Nurses	33 (22.8)	24 (25)
Service and support staff	20 (13.8)	12 (13)
Dispatch Centre	2 (1.4)	1 (1)
Prehospital staff	6 (4.1)	2 (2)
Police	2 (1.4)	2 (2)
Rescue service	2 (1.4)	1 (1)
Military	2 (1.4)	0 (0)
Counterplay	26 (17.9)	13 (14)
Instructors	15 (10.3)	11 (12)
**Total**	145 (100)	95 (100)

### Resource staff

A counterplay was set up in both exercises with representatives from strategic command (the regional level), EMS dispatch center, property management, police authority and rescue service. The strategic command deals with the mobilisation and allocation of resources in the region. In Exercise 1, the participation was extended with representatives from public transport, county administrative board, municipality of Stockholm, and the Swedish armed forces. Prior to Exercise 1, the upcoming exercise was presented at national, regional, and local levels. Several planning meetings and regional activities took place to identify possibilities for cooperation and request support from external organisations not normally associated with the regional authority during a major incident, such as non-governmental organisations.

### Planning

Each exercise required considerable planning. The simulation system provided detailed information on resources and procedures that the hospital staff, EMS and collaborating agencies needed to carry out in the event of an evacuation. Hospital staff carried out an inventory of typical patients in hospital at a time and day of the week corresponding to the planned exercise. Inventory was made to define:

- number of in-hospital and ambulatory patients.
- information on continued need for care (level of care).
- whether the patient could walk on his own or with support, needed a wheelchair, or recumbent transport/stretcher transport. It also declared the possible need for medication, isolation, interpreters, and crisis support. This information was then registered on the patient card that formed the basis for triage and evacuation during the exercise.
- number of hospital staff from different categories available at particular points in times.
- which hospitals units considered critical in the event of water supply failure: ICU, surgery, laboratories etc.
- how many evacuated patients that could be relocated in the region.
- transport resource categories in the region, availability, and within what time frame.

### The simulation system

**Fig. 1:**
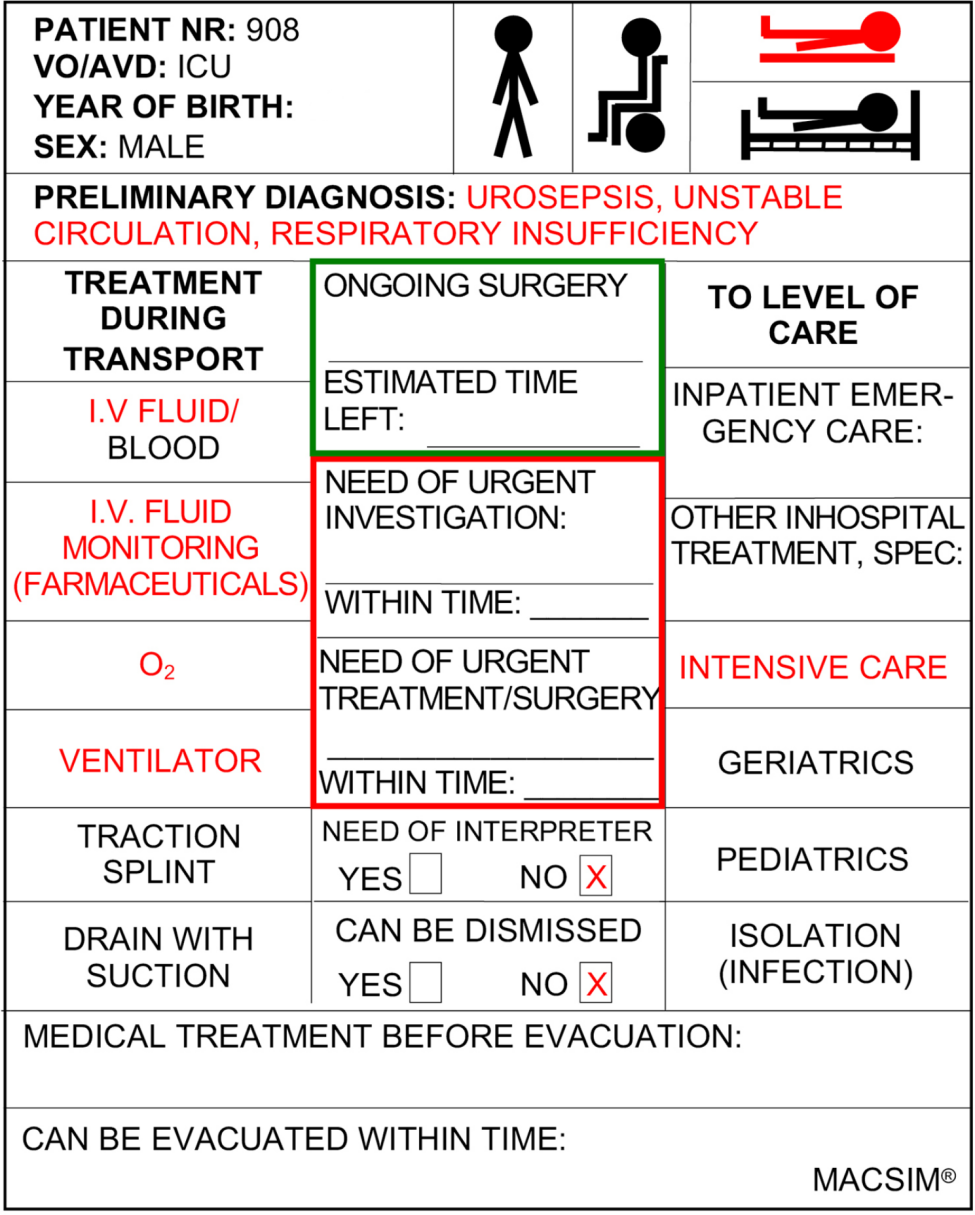
Patient card for evacuation exercise

Based on the inventory, laminated plastic cards were prepared for all fictive inpatients and outpatients theoretically in the hospital when the exercise began (Fig. 1). Use of personal data was strictly prohibited. The card number was used as patient ID. The options that applied to each patient were marked in red. These cards revealed the following medical data: diagnosis, need of care, transport needs, need of treatment/monitoring during transport and need for urgent examination or treatment within a certain time after evacuation. Colored post-it notes were used as support during triage, being attached to the cards with the level of care marked. The different colors indicated the following:

- Red tab: Patients requiring the highest level of care.
- Yellow tab: Patient requiring a lower level of care.
- Green tab: Patients who could be discharged from the hospital.

### Facilities for the exercise

**Fig. 2:**
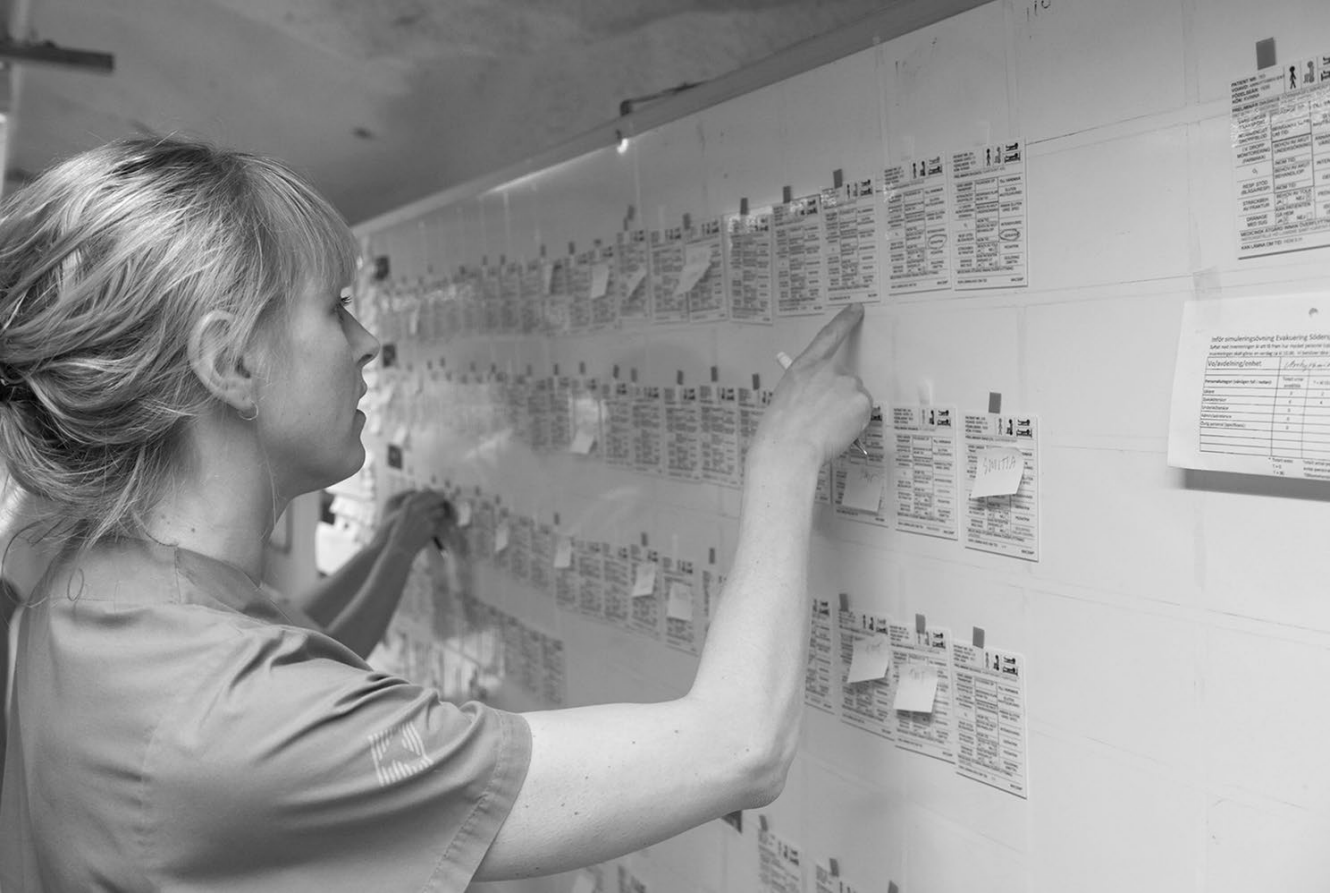
Unit staff gathering and reporting data for each patient

Staff involved in the exercises worked together at whiteboards. Each unit and ward were provided with whiteboards illustrating all their admitted patients. Staff performed inventory and preparing patient transports etc. (Fig. 2).

**Fig. 3:**
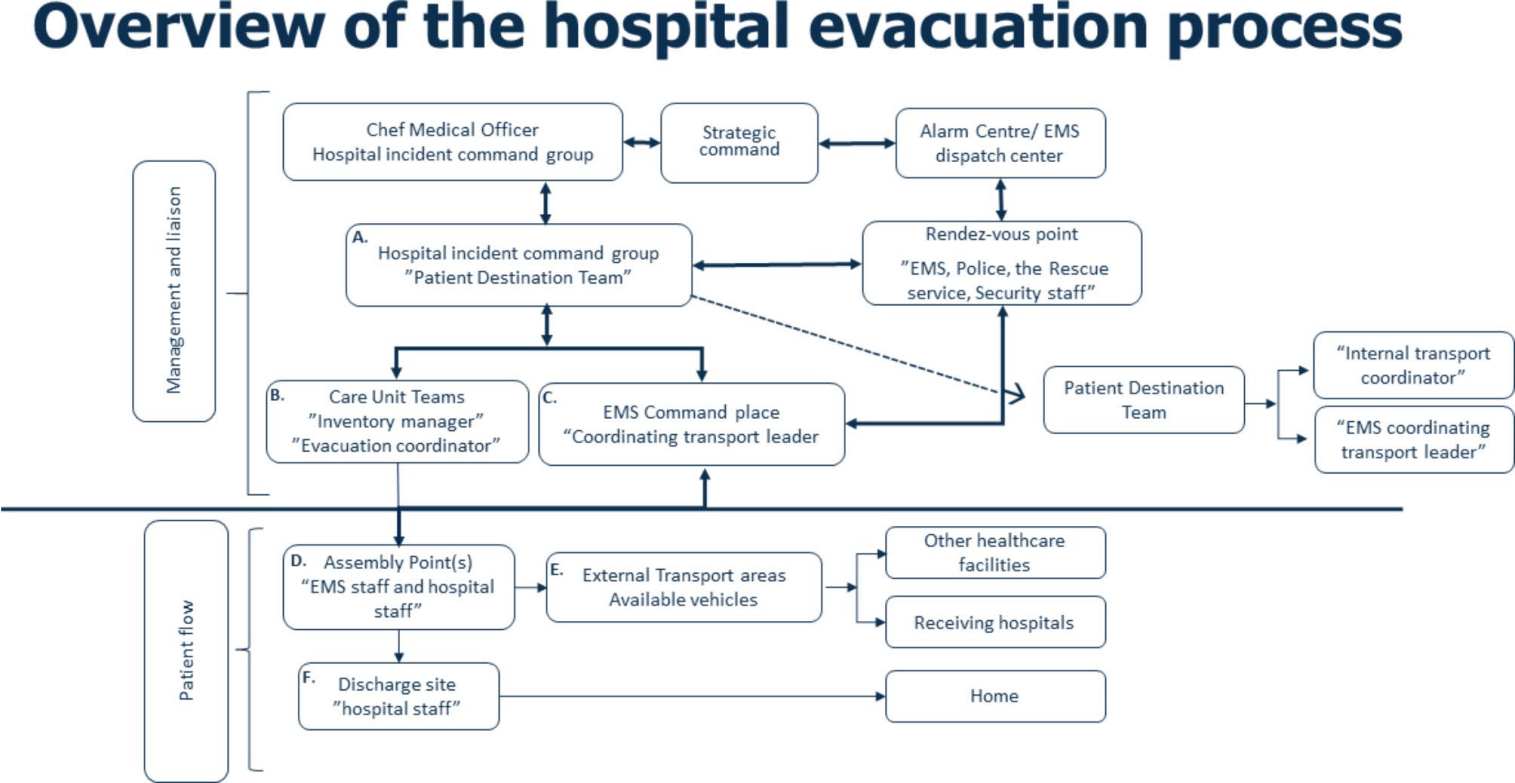
Overview of the hospital evacuation process

**Fig. 4:**
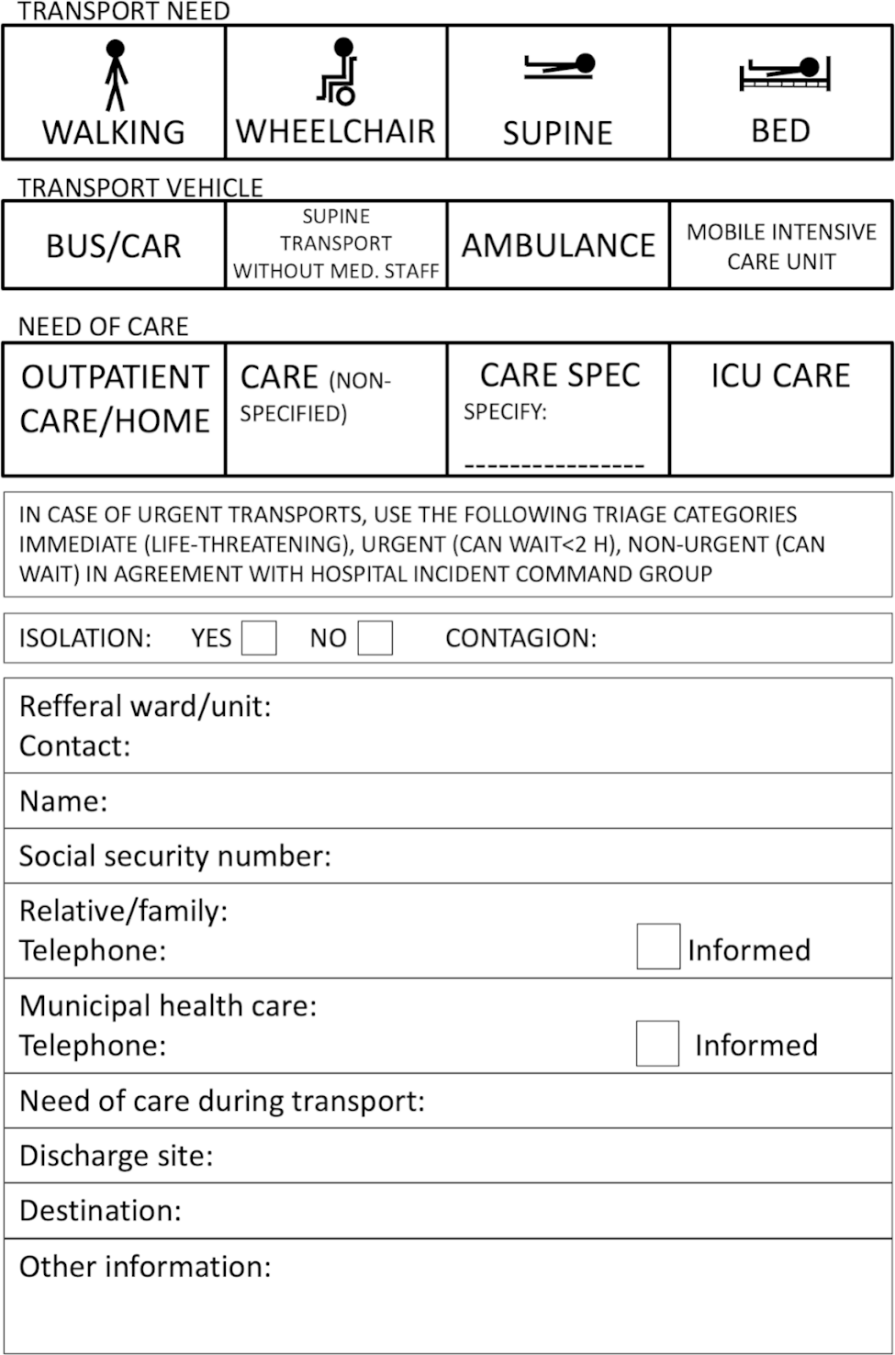
Information card evacuation

**Fig. 5:**
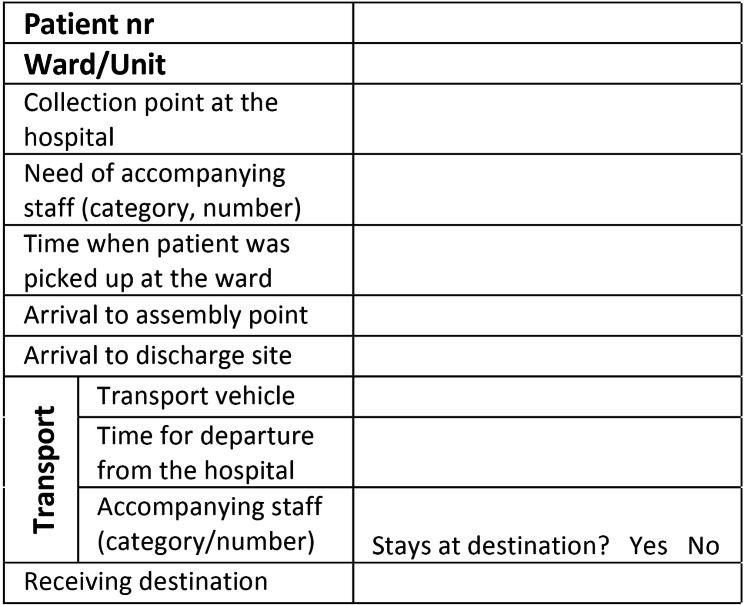
Self-adhesive marker patches

The time required to move patients, staff, and material had been calculated in advance. Several protocols and forms were produced, in combination with the evacuation plan. Using these protocols, participants were able to accurately determine patient need for care, patient departure checklist, patient transport to assembly point (AP) and discharge site (DS), and requirements of transport to the receiving facility etc. Once the decision to evacuate was made, following specific functions were activated to support HICGs work with the evacuation process (Fig. 3): **A**) “Patient Destination Team” is responsible for transport logistics and information to units about where, how, and when the patients are to be moved to the loading area. **B**) “Care Unit Teams” each care unit prepare the discharge of patients (medications, medical records, necessary equipment, and transport needs), managed by a “Unit Leader” in collaboration with responsible clinicians and their Evacuation Coordinator. **C**) “EMS command place” is responsible for management and coordinating different types of ambulance transport. **D**) “Assembly Point” is staffed by EMS and hospital staff responsible for registration and patient care until patients are ready to be transferred to the transport loading area. **E**) “External transport areas” in this area staff register and confirm patient identity and transfer destination before loading patients into an ambulance. **F**) “Discharge site” is staffed by hospital staff to log and supervise patients while waiting for transport to their home. To keep track of the patient and as a back-up system for information regarding the patient, pre-printed forms and information cards were used (Figs. 4 and 5).

**Fig. 6:**
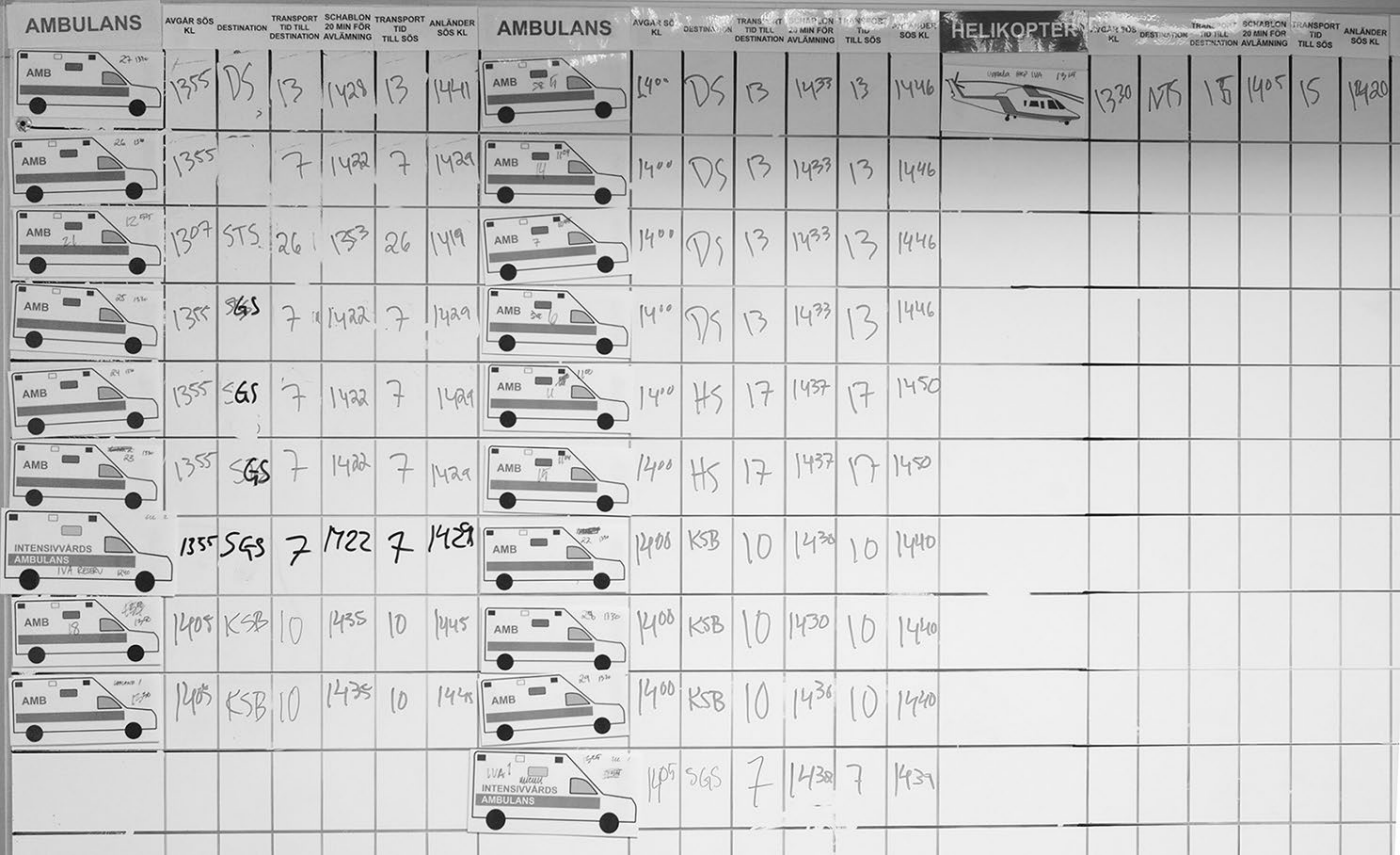
Whiteboards showing requested transport vehicles

Those responsible for transport worked with ambulance staff at whiteboards where requisitioned transport vehicles were shown as moveable magnetic symbols, and estimated times of arrival were registered according to the pre-planned transport times (Fig. 6). All transport vehicle symbols placed on the whiteboards had information from the arrival time at the evacuating hospital to the arrival time at the receiving facility and return. The objective was to maintain patient safety when assigning transport vehicles, care teams and receiving beds, given the available resources. Each training station was assigned communication devices.

### Data collection

The exercises were evaluated regarding evacuation factors, organisation, and exercise methodology. All participants were asked to answer an anonymous post-exercise survey and, depending on their role, they answered specific questions concerning the evacuation process. The surveys were carried out immediately after each exercise. To capture degree of agreement, a 10-point Likert scale was used. Score of 1 indicated less favorable (very poor) and score of 10 indicated most favorable (very good). The survey also allowed the respondents to give feedback in open-ended questions that helped uncover other details and provided a more complete picture of the evacuation process. The questions included: How would you describe your experience of the evacuation process at your unit? Based on your experience from this exercise, do you have any views on the management, coordination, and communication, and how this could be improved? What would you like to change or add to the evacuation plan?

### Statistical analyses

The survey data were entered into Microsoft 365 Excel spreadsheet (Microsoft Corporation, Redmond, Washington, USA) and are described as mean, standard deviation (SD) and percentages. A qualitative content analysis was performed (guided by the COREQ checklist) using systematic text condensation [26]. The method contains a four-step procedure. First (1) the text is read to capture themes, then (2) code units of meaning are extracted and (3) condensed into core contents and finally (4) the findings are summarized.

### Ethical considerations

The study complied with the Helsinki Declaration on research ethics [27]. No approval was required from ethical committees since data were collected anonymously as participants and patient identification was never revealed in the post-exercise surveys or patient cards.

## Results

### Findings based on registration of patients and utilised resources

From the time when hospital evacuation was initiated until the exercise was stopped, 683 (71.2%) of 959 patients in seven hours (exercise 1) and 234 (52.9%) of 442 patients in four hours (exercise 2) were evacuated. Specific data regarding patients assessed to need assistance for transfer, and number of patients evacuated by transport vehicles during the exercises are presented in Tables 2 and 3.

**Table 2:** Patients’ assessment for transfer

	Exercise 1	Exercise 2
Assessed need	n (%)	n (%)
No assistance, walking	532 (55)	164 (55)
Wheelchair	119 (13)	81 (18)
Supine transport (stretcher)	178 (19)	112 (26)
ICU transport	19 (2)	5 (1)
Newborn and children < 2 y	79 (8)	0 (0)
Not assessed	32 (3)	0 (0)
**Total**	952 (100)	442 (100)

**Table 3:** Number of patients evacuated by transport categories during the exercises

	Exercise 1 Time: between 9.52 am and 4.00 pm	Exercise 2 Time: between 1.10 pm and 4.00 pm
**Type of vehicle**	n (%)	n (%)
Ambulance	72 (10.5)	3 (1)
ICU transport	5 (0.7)	3 (1)
Helicopter	14 (2.1)	0 (0)
Transport service (nonurgent medical transport)	76 (11.1)	0 (0)
Bus	15 (2.2)	0 (0)
Taxi	90 (13.2)	30 (13)
Police transport	1 (0.2)	0 (0)
Private transports	410 (60)	198 (85)
**Total**	683 (100)	234 (100)

### Findings or lessons learned based on the post-exercise survey

A total of 64 participants replied in exercise 1 (out of 145 = 41.1%) and 68 in exercise 2 (out of 95 = 72%). Table 4 shows the self-assessment result for the surveys completed expressed as mean, standard deviation (SD) and percentage. The survey showed that 1.6% and 3.0%, respectively, of the respondents had experience of evacuating a hospital during a real situation. On the question if the exercise gave them a fair view of how the hospital would cope with an evacuation under given conditions, the respondents answered with a mean of 7.5 (SD 1.8) and 7.6 (SD 1.4) respectively. Regarding the findings about the problems that might occur during an evacuation and how to deal with them, the respondents answered with a mean of 7.9 (SD 1.7) and 8.0 (SD 1.5) respectively. On the question if the simulation system fulfilled the function of illustrating and training hospital evacuation, the respondents answered with a mean of 7.7 (SD 1.8) and 7.9 (SD 1.4) respectively. Furthermore 97% and 95%, respectively, thought it would be worthwhile to have more exercises of this kind. The survey also addressed questions regarding the function of the evacuation plans, organisations, and hospital management. On the question if the hospitals proposals for evacuation plans tested during the exercises fulfilled its function, the respondents answered with a mean of 7.2 (SD 1.7) and 7.2 (SD 1.7) respectively.

**Table 4:** Results of post-surveys. The results are described as mean, SD and percentage

Questions	Exercise 1/ Exercise 2	n	(%)	Mean (SD)
**Questions regarding the exercise methodology** answered by all participants (Exercise 1 n = 145, Exercise 2 n = 95)				
How do you assess the written information you received before the exercise?	1	61	42	7.2 (2.0)
	2	65	68	7.8 (1.9)
How do you assess the value of the workshops that were arranged before the exercise?	1	32	22	7.5 (1.9)
	2	22	23	7.9 (1.7)
Do you think that the exercise gave you a fair view of how the hospital will cope with an evacuation under the given conditions?	1	58	40	7.5 (1.8)
	2	66	69	7.6 (1.4)
Did you learn anything about the problems during an evacuation and how to deal with it, thanks to the exercise?	1	63	43	7.9 (1.7)
	2	64	67	8.0 (1.5)
Do you feel that the simulation system used fulfills the function of illustrating and training hospital evacuation?	1	52	36	7.7 (1.8)
	2	55	59	7.9 (1.4)
**Questions regarding the evacuation plan, organization, and hospital management** answered by hospital staff (Exercise 1 n = 90, Exercise 2 n = 65)				
Do you think that the hospitals´ proposal for evacuation plan, tested during this exercise, fulfils its function?	1	40	44	7.2 (1.7)
	2	55	85	7.2 (1.8)
How do you assess the organization of the evacuation of your unit?	1	42	47	8.4 (2.0)
	2	40	61	7.4 (2.0)
**Questions** answered by hospital and EMS staff as well as actors in the counterplay (Exercise 1 n = 122, Exercise 2 n = 80)				
How do you assess the information from HICG?	1	36	29	6.1 (2.4)
	2	13	16	5.1 (3.5)
How do you assess the HICGs management ability?	1	34	28	6.4 (2.3)
	2	13	16	6.2 (2.5)
**Questions** answered by the Hospital Incident Command Group (Exercise 1 n = 24, Exercise 2 n = 16)				
How do you assess the information you received from the various units?	1	6	25	6.7 (1.5)
	2	13	65	7.6 (1.3)
How do you assess the collaborative command and control with assembly points/ discharge site?	1	18	75	7.1 (2.2)
	2	11	55	6.5 (1.4)
How do you assess the collaboration with regional medical command group?	1	8	33	6.7 (2.4)
	2	11	55	7.3 (1.3)
How do you assess the collaboration with other external actors?	1	10	42	7.6 (1.3)
	2	13	65	7.5 (1.5)
**Question** answered by resource persons (Exercise 1 n = 35, Exercise 2 n = 23)				
How do you assess the amount of information from the exercise management before the exercise?	1	17	49	7.5 (2.3)
	2	17	74	8.6 (1.5)

### Qualitative data

Open-ended questions contributed a qualitative element to the quantitative survey. A qualitative content analysis of the open-ended questions defined five main categories to take into consideration when planning detailed evacuations: (1) Management and liaison; (2) Communication; (3) Logistics; (4) Medical care and patient prioritisation; and (5) Resource utilisation.

### Management and liaison

In this category, accurate decision-making, leadership, resource management, triage priority, and information sharing were considered vital components in successful management. All staff gained valuable experience regarding the difficulties involved in medical decision-making, coordination, and collaboration between unit managers, patient destination team, and strategic command.

### Communication

Communication proved to be a challenge, exposing the need for better procedures regarding sharing of information. The unit staff felt that real-time updates from the HICG on transport times and priorities for inpatients was insufficient and that this made patient transfer suboptimal. Strategies designed to spread information and decisions that must be implemented rapidly, to staff, patients, and family members, was requested.

### Logistics

Several weaknesses in the internal and external logistic plans were identified by the staff. These included lack of a clear role, complicated registration forms, and unnecessary time waiting for transport, all of which caused frustration. One other issue mentioned was the handling of patients and relatives gathered in the main entrance. This could potentially lead to need of acute medical care and problems in maintaining order.

### Medical care and triage

Unit staff perceived insufficient information on how the HICG had broadly envisioned the evacuation process to be carried out. They felt that the plan should have stated the specific units to be given immediate priority in the early stages, and that subsequent order of priority should follow response and triage methods based on the state of events. The staff underlined the importance of the medical technician’s role in the evacuation plan due to the risk of losing or damaging medical equipment during transport to the receiving facility.

### Resource utilisation

The need for specific staff reinforcement in terms of resources at the assembly point and administrative staff both at care units and the command center was highlighted. There was a need for a strategy for relocation of staff as evacuation progressed, particularly the assignment of extra staff. In both exercises it became clear that coordination of internal and external transport was crucial for the evacuation process.

## Discussion

### Discussion of findings

These results demonstrate how a real-time simulation system can help emergency planners to develop, test and contribute to the revision of evacuation plans by identifying shortcomings of those currently in place (if any). Training combined with simulation exercises increase staff skills and strengthen preparedness and resilience to any future disaster [4, 12, 17, 28, 29].

During the exercises, the hospital bed capacity in Region Stockholm was not overwhelmed and evacuation went smoothly. Patient allocation to receiving facilities was directly linked to resource availability (e.g., ICU beds, ambulances, transport units) and were coordinated by the strategic command and the EMS dispatch center. In this moderately urgent scenario, results indicate that the HICG’s time frame for complete evacuation was feasible in both exercises (24 h). Delay in decisions on how to use resources in the most efficient way may prevent timely evacuation. Review of the literature shows that collaboration, coordinated decision making, and communication are major contributors to success or failure of hospital evacuation [5, 11]. Prior experience from multiple case reports shows that shared situational awareness is a key component of efficient communication and that disaster plans should anticipate the failure of communication systems [15, 30].

Lack of situational awareness proved to be a challenge throughout the exercises, and inadequate communication between and within care units and the HICG impeded evacuation efforts which is reflected in the survey responses. During hurricane Sandy, nurses described the importance of support from their chiefs as well as continuous information from other units, enabling them to better fulfill their tasks [28]. In this study, participants expressed similar thoughts. From the staff perspective, support from colleagues, personal resourcefulness, giving support to others, and leadership qualities are citated as crucial factors when carrying out their roles.

The simplicity of the evacuation process was an important issue in the planning phase of the exercises. This requires an intuitive plan that is easy for staff to understand and act upon. The task for hospital unit staff was to prepare patients for safe transfer. Preparation of medical information, equipment and medications that were to accompany patients to their new destination as well as the assurance of adequate staffing was the first stage in the evacuation process. Checklists prior to patient departure, gathering and reporting data for each patient, were used to inform and assist clinicians at the receiving facility. The second stage was to ensure that all patients were registered and transported, and that family members or other relatives were notified about the patient’s transfer destination.

The HICG was flooded with information making it difficult to gain an overview of patient distribution and transport resources available. Furthermore, in both exercises the coordinating transport leader perceived communication as burdensome. Lack of communication created bottlenecks in the ambulance flow (specially in exercise 2, see Table 3), but ambulance capacity was not a limiting resource since there were more ambulances than needed. The process of managing ambulances in exercise 2 was disorganised due to lack of communication between HICG and EMS command place. Review of the literature shows that it is unlikely that an increase in ambulance resources will reduce the duration of evacuation [13]. Time required for identifying patient destination and other factors that could lead to delayed departure were not calculated in advance since this was mainly handled by the counterplay.

ICU patients who required skilled nursing care were difficult to transfer because arrangements took time to coordinate. In general, the literature recommends evacuating the most resource-intensive patients first [30]. However, data on best practice are limited. Deciding priority when moving patients was based on the amount of time and transport resources available. By using patient cards (Fig. 1) with actual patient information and forms developed for individual inpatient care units, each unit could test and evaluate the protocol toolkit during the evacuation. However, the time requirements for preparing patient documentation were not calculated in the exercises. Before departure from each unit, instructors followed up that requested information was filled in. Use of practical “tools” that guide staff during an evacuation is essential and must be developed and tested [31]. Protocols were followed, but the manual tracking system (with selected “check-in” and “check-out” data at several checkpoints) posed a logistic challenge for the hospitals during the exercises. It was difficult for the patient destination team to gather data and gain an overview of patient location during evacuation. The scenario was chosen to test the logistics in a less-urgent situation. Time still became a critical factor since it was important not to keep dispatched and designated transport resources waiting.

Furthermore, the use of manual registers was perceived as stressful. In the future, automated system may be more effective for information sharing and less stressful in the event of disaster [32]. Electronic tracking system using barcodes on patients’ armbands, scanned with tablets or smartphones with a dedicated app should easily be able to overcome these challenges.

Evacuation is a labor-intensive process, and the Human Resources Department should be activated as soon as possible. The theoretical inventory predicted the numbers of general and clinical staff at the time the exercise began and the next following 12 h. When the inventory was made, the hospitals had to take into consideration how much staff of each category they would be able to alert and activate during the different levels of preparedness. When the level of disaster was declared during the exercises, the staff was arriving on given times according to the inventory. In both exercises, the availability of extra staff, according to the regular schedule, was estimated to be sufficient as the day progressed, given that many patients had already left the hospital. However, there was no clear structure for assessing staff endurance and provision of relief. One important lesson regarding the evacuation process was the need to have mobile care resource teams at several checkpoints ready to respond to care needs along the evacuation route.

Effective training and exercises are important cornerstones in disaster preparedness and response [33]. The simulation system used in this study, was shown to be useful in testing and revising hospital evacuation plans. Participants felt the system largely fulfilled its function of mimicking an evacuation and providing process training. Implementation of realistic exercises is a complex task requiring significant work efforts of those who are responsible in terms of resources as workforce, commitment, and cost [4]. The exercises could be carried out at a limited cost as interference with ongoing medical care was minimal. The use of structured simulation models makes it easier for emergency planners in planning for evacuation of hospitals and develop workable evacuation plans. Experience from the exercises revealed that there is more work to be done to revise evacuation plans, especially facilitating communication, simplifying specific evacuation protocols and registration forms, as well as improving coordination between various functions and transport teams. This kind of system increases our understanding of the evacuation process, management and decision making, ultimately leading to better outcome during a real hospital evacuation. To achieve adequate evacuation preparedness the need for “table top” exercises using a validated simulation system for hospital emergency planners is both essential and necessary.

### Limitations/methodological considerations

This was the first time the simulation tool was used in order to test an evacuation scenario to this extent. The generalisability of our findings may be limited because the exercises differed slightly in their structure and the survey response rates were low. According to Malterud et al. [34] a lower response rate is acceptable if the participants are knowledgeable, specifically selected for the aims of the study and the answers are comprehensive. Systematic text condensation does not differ significantly from existing content analysis methods, and there is always the risk of fragmentation of data and loss of information if the amount of data is limited. However, it is likely that evacuation planning and procedures for different care units can be generalised and used within the hospital and at different hospitals. Treatment during transport and on arrival at the receiving care facility were not included in this study.

## Conclusions

This study has shown that “table top” exercises using a validated simulation system can serve to guide emergency planners when developing evacuation plans, procedures, and protocols as well in training of all medical staff. The simulation system revealed shortcomings in currently used evacuation plans and identified several key areas to improve emergency preparedness. The system also served to train adaptive thinking, leadership, communication, and clarification of critical functions.

## Abbreviations

AP: Assembly Point
DS: Discharge Site
HICG: Hospital Incident Command Group
EMS: Emergency Medical Services
